# *Viola kauaensis* var. *hosakae* (Violaceae), a new variety of endemic Hawaiian violet

**DOI:** 10.3897/phytokeys.39.6500

**Published:** 2014-06-23

**Authors:** J. Christopher Havran, Susan Ching Harbin, Talia Portner

**Affiliations:** 1 Department of Biological Sciences, 205 Day Dorm Rd, Campbell University, Buies Creek, NC 27506, USA; 2 O`ahu Plant Extinction Prevention Program, 2551 Waimano Home Rd, Rm 202, Pearl City, HI 96782, USA

**Keywords:** Hawaiian Islands, O`ahu, Violaceae, *Viola kauaensis* var. *hosakae*, cleistogamy

## Abstract

The Hawaiian endemic *Viola kauaensis* A. Gray has a broad distribution in bogs of Kaua`i and a limited distribution on mesic ridges in the Ko`olau Mountains of O`ahu. Based on differences in scale, the O`ahu populations of *Viola kauaensis* had previously been described as a distinct taxon. The taxonomic status of the O`ahu populations was reevaluated through a morphometric analysis of all varieties of *Viola kauaensis* and the morphologically similar *Viola vanroyenii*. Morphological features of historic and freshly collected specimens of all varieties of *Viola kauaensis* were analyzed with a principal components analysis. Populations from O`ahu represent a distinct cluster that slightly overlaps with *Viola kauaensis* var. *kauaensis*. Lamina width, apex angle, and base angles contribute to the separation of the O`ahu populations from other varieties of *Viola kauaensis*. Due to differences in scale, the O`ahu populations are described as *Viola kauaensis* var. *hosakae*, a new critically endangered taxon.

## Introduction

*Viola kauaensis* A. Gray is one of nine species of the monophyletic Hawaiian violets (Violaceae) (Ballard and Sytsma 2000, Havran et al. 2009). The species grows from a creeping rhizome and is notable among endemic Hawaiian *Viola* in being both herbaceous and bears cleistogamous flowers (Wagner et al. 1999). Two varieties of *Viola kauaensis* are recognized: *Viola kauaensis* var. *kauaensis* possesses rotund to cordate leaves and is distributed primarily in high elevation bogs and cloud forest margins in central to northwestern Kaua`i (Wagner et al. 1999); *Viola kauaensis* var. *wahiawaensis* Forbes is distributed in the Kanele (Wahiawa) Bog and nearby ridges north of the town of Hanapepe, Kaua`i (Forbes 1920, Wagner et al. 1999). *Viola Viola kauaensis wahiawaensis* can be differentiated from *Viola kauaensis* var. *kauaensis* by cuneate leaf bases (Forbes 1920, Wagner et al. 1999). While *Viola kauaensis* var. *kauaensis* is locally abundant, *Viola kauaensis* var. *wahiawaensis* is a federally listed endangered taxon.

Additional populations of *Viola kauaensis* are located in the Ko`olau Range on the neighboring Hawaiian Island of O`ahu. Individuals in the O`ahu populations resemble *Viola kauaensis* var. *kauaensis* but possess smaller leaves, stipules, flowers, and fruits (St. John 1989). *Viola kauaensis* is rare on O`ahu and is distributed on sloping exposed or mossy ground, not in open bogs or forest margins like on Kaua`i. No evidence has been found that the populations on O`ahu produce cleistogamous flowers. Fosberg and Hosaka (1938) mention that “the specimens from O`ahu correspond very well with the dwarf form from the bogs” of Kaua`i. Due to differences in scale, the individuals in the O`ahu populations were named *Viola hosakae* St. John by St. John (1989) in a systematic treatment of all Hawaiian *Viola*. The O`ahu specimens of *Viola kauaensis* were not available for study during the drafting of *Manual of the Vascular Plants of Hawai`i* (Wagner et al. 1999) and were therefore not treated in that publication. In *The Flora of the Hawaiian Islands* website, *Viola hosakae* is placed in synonymy with *Viola kauaensis* var. *kauaensis* but W. Wagner noted: “probably this should be treated as a third taxon of *Viola kauaensis*”.

St. John (1989) named one other violet that is morphologically similar to *Viola kauaensis*. *Viola vanroyenii* St. John represents a population of small herbaceous violets endemic to the summit area of Mt. Wai`ale`ale on Kaua`i. One collection was made from the summit by van Royen and Perlman in 1977 (P. van Royen 11733 [BISH]). The population is represented by one herbarium sheet at BISH containing 14 individual plants and several fragments. The species is distributed within the range of *Viola kauaensis* var. *kauaensis* on Kaua`i and has yet to be rediscovered (Ken Wood and Steve Perlman, personal communication).

In the current study, the taxonomic status of O`ahu populations of *Viola kauaensis* was reevaluated through an analysis of vegetative and reproductive traits of all varieties of *Viola kauaensis* and of *Viola vanroyenii*. We asked three questions: (1) Is variation in morphological traits discontinuous between interisland populations of *Viola kauaensis*?; (2) Do the O`ahu populations of *Viola kauaensis* produce cleistogamous flowers?; and (3) Should the O`ahu populations of *Viola kauaensis* be treated as a distinct taxon?

## Methods

### Field collection

*Viola kauaensis* has been documented from several sites on O`ahu. The first recorded collection of the species in 1938 was recorded as “Ko`olau Range, divide between head of Kawainui and Kaipapau Gulches” (*E.Y. Hosaka 2,504* [BISH]). The site has not been relocated since the original collection. Two additional populations are located in the Poamoho region of the Ko`olau Mountains (due to the rarity of the species, the population locations are referred here as sites A and B). Both locations were visited in May 2013 to make new collections and assess the size of populations.

Site A is located near the Poamoho Trail and contains four small subpopulations. Because the species is considered locally threatened only two whole individuals were collected. One additional flower was collected and preserved in 70% ethanol for dissection. The fragmented nature of the populations and their position on nearly vertical cliff faces (which appears typical for individuals on O`ahu) prevented a random assessment of individuals from the site. To obtain a measure of the size of individuals at Site A, the length and width of the largest lamina of several individuals was measured. A 6 m transect was run parallel to the summit of one ridge. A 2 m tape was extended down the slope of the cliff every 1 m. Individuals easily accessed within 50 cm of the tape were measured.

Site B is located along the Ko`olau Summit Trail. The population was discovered in 1986 by John Obata. Information on the population was shared with Clyde Imada of the Bishop Museum Herbarium (personal communication). The site was revisited by Clyde Imada in 1995 who did not observe any *Viola* at that time. In May 2013, Site B was revisited to survey for *Viola kauaensis*. No individuals of *Viola kauaensis* were rediscovered at Site B.

Label data from the type of *Viola vanroyenii* collected in 1977 (*van Royen 11733*) indicates that all specimens of *Viola vanroyenii* were collected from the “summit area of Mt. Wai`ale`ale”. In November 2012, the summit of Mt. Wai`ale`ale was visited by Kyle Kagimoto (The Nature Conservancy of Hawai`i). Five samples of *Viola kauaensis* were made from the summit area and are included in the current study.

### Measurements

All specimens representing varieties of *Viola kauaensis* and *Viola vanroyenii* on deposit at BISH and DUKE herbaria were analyzed. Digital scans of O`ahu specimens of *Viola kauaensis* from PTBG were examined. The type specimens of *Viola kauaensis* var. *wahiawaensis*, *Viola vanroyenii*, and *Viola hosakae* were analyzed at BISH. A digital scan of the type of *Viola kauaensis* from US was analyzed. Five specimens of *Viola kauaensis* collected by Kyle Kagimoto were deposited at CAU. Morphological variables measured include: length and width of the leaf lamina, cauline stipules, rhizome stipules, and sepals; length of petioles and capsule valves; and apex and base angles of the leaf lamina. Very few specimens of *Viola kauaensis* from O`ahu possessed intact or fully developed petals. Therefore we restricted our floral measurements to sepal characters only. The largest leaf on each specimen was chosen for measurements of foliar characteristics. If a leaf was folded, damaged, or wrinkled to the extent that it could not be determined if it represented the largest leaf, the next largest leaf was chosen for measurement. All size measurements were made to the nearest 0.5 mm. Apex angle was measured as the angle of two rays running along the margins of the leaf tip with the vertex placed just above (at or within 1 mm) of the leaf tip. Base angle was measured as the angle of two rays running along the base of the lamia with the vertex placed just below (at or within 1 mm) of the tapered base of the lamina (Ellis et al. 2009). Some specimens of *Viola kauaensis* var. *kauaensis* possessed cordate leaf bases. In these cases, base angle was measured as the angle of two rays running along the inner margins of the left and right portions of the reniform base with a vertex placed at the insertion point of the lamina (Ellis et al. 2009). For one folded leaf of *Viola kauaensis* var. *wahiawaensis*, base angle was estimated based on one half of a folded leaf.

Only one sheet of *Viola vanroyenii* exists at BISH. The sheet contains 14 individual stems and several fragments. We attempted to measure as many entire samples from this sheet as possible to obtain a robust estimate of morphological variation in the taxon. Only three individuals on the sheet possessed all traits required for a principal components analysis.

Multiple individuals were measured from herbarium sheets when it appeared that vegetative and reproductive structures were attached to separate rhizomes. Digital images of herbarium specimens were used where possible. ImageJ software (Rasband 2012) was used to analyze digital images.

### Analyses

A principal component analysis (PCA) was used to investigate the morphological variation between interisland populations and varieties of *Viola kauaensis*. The PCA was conducted with varimax rotation on untransformed data. Many herbarium specimens contained samples with degraded, fragmented, missing, or not-otherwise obvious characteristics. Therefore, only specimens that had complete measurements for lamina length, lamina width, petiole length, apex angle, base angle, cauline stipule length, and cauline stipule width were included in the analysis. The 49 samples incorporated into the PCA are listed in Table 1. In accordance with the Kaiser rule, principal component (PC) loadings with eigenvalues above 1.0 were retained for further analysis. Analyses were conducted in R version 2.15.1 (R Core Team 2012).

**Figure 1.**
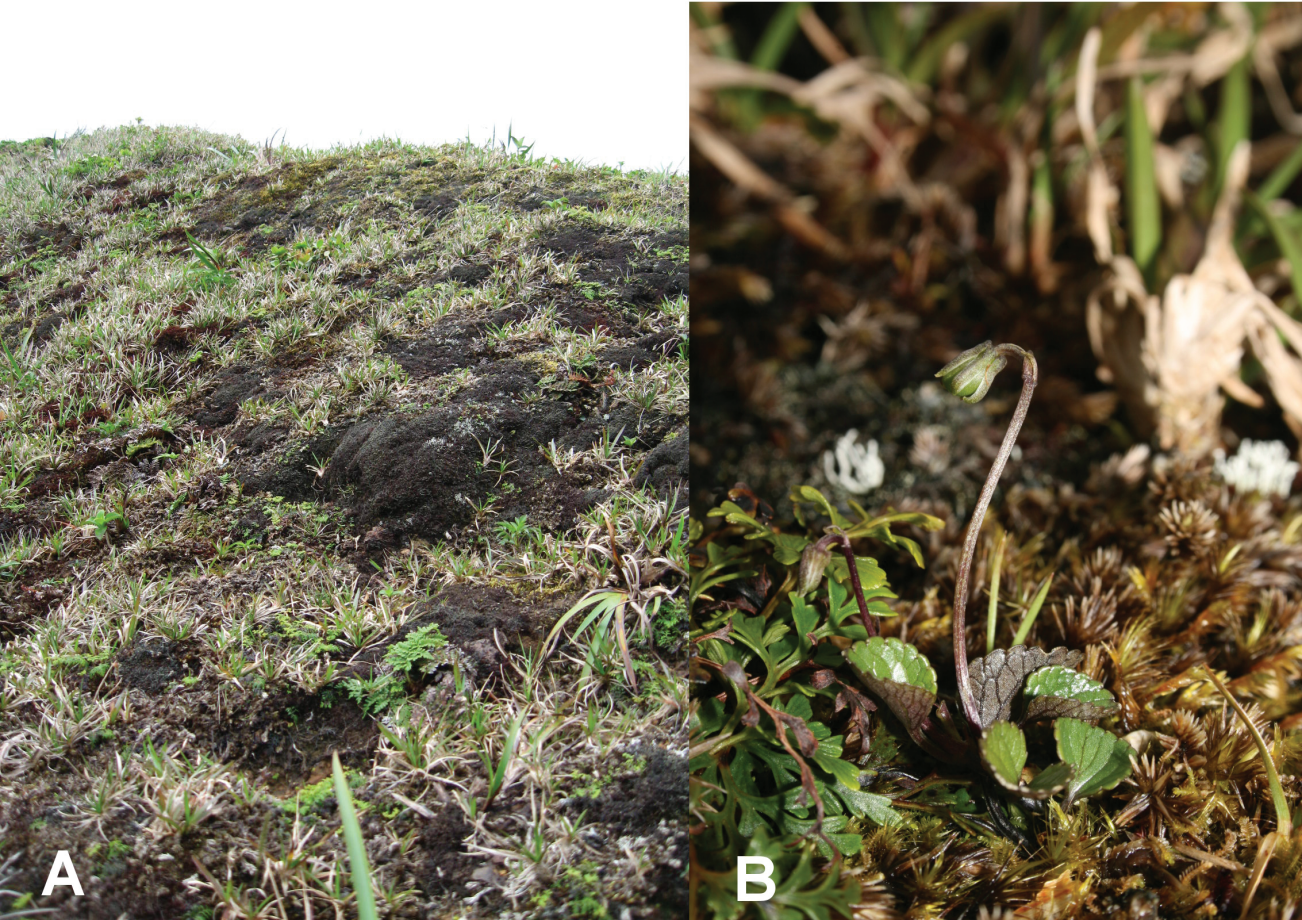
*Viola kauaensis* var. *hosakae* on O`ahu. **A** Habitat composed of mossy slope **B** Individual in fruit. (Photo credits: **A** J. C. H., **B** Joel Lau).

**Table 1.** Samples incorporated into Principal components analysis.

**Taxon**	**Samples per sheet**	**Island**	**Collection No.**	**Herbarium**
*Viola kauaensis* var. *hosakae*	3	O`ahu	*E.Y. Hosaka 2504*	BISH
*Viola kauaensis* var. *hosakae*	1	O`ahu	*E.Y. Hosaka 1927*	BISH
*Viola kauaensis* var. *hosakae*	1	O`ahu	*F.R. Fosberg 13973*	BISH
*Viola kauaensis* var. *hosakae*	1	O`ahu	*F.R. Fosberg 14229*	BISH
*Viola kauaensis* var. *hosakae*	1	O`ahu	*J.C. Havran 2013.4*	BISH
*Viola kauaensis* var. *hosakae*	1	O`ahu	*J.C. Havran 2013.5*	BISH
*Viola kauaensis* var. *hosakae*	1	O`ahu	*S. Perlman 14704*	PTBG
*Viola kauaensis* var. *kauaensis*	2	Kaua`i	*C.N. Forbes 1135K*	BISH
*Viola kauaensis* var. *kauaensis*	1	Kaua`i	*C.J.F. Skottsberg 939*	BISH
*Viola kauaensis* var. *kauaensis*	1	Kaua`i	*J.C.F. Rock 2124*	BISH
*Viola kauaensis* var. *kauaensis*	1	Kaua`i	*J.C.F. Rock 2130*	BISH
*Viola kauaensis* var. *kauaensis*	1	Kaua`i	*C.N. Forbes 906 K*	BISH
*Viola kauaensis* var. *kauaensis*	1	Kaua`i	*D.R. Herbst 2388*	BISH
*Viola kauaensis* var. *kauaensis*	1	Kaua`i	*P. van Royen 11708*	BISH
*Viola kauaensis* var. *kauaensis*	1	Kaua`i	*O. Degener 21747*	BISH
*Viola kauaensis* var. *kauaensis*	3	Kaua`i	*H. St. John 10753*	BISH
*Viola kauaensis* var. *kauaensis*	1	Kaua`i	*C.N. Forbes 406 K*	BISH
*Viola kauaensis* var. *kauaensis*	1	Kaua`i	*J.F.C. Rock 2131*	BISH
*Viola kauaensis* var. *kauaensis*	1	Kaua`i	*O. Degener 21477*	BISH
*Viola kauaensis* var. *kauaensis*	2	Kaua`i	*H. St. John 23038*	BISH
*Viola kauaensis* var. *kauaensis*	2	Kaua`i	*W.N. Takeuchi Alakai_130a*	BISH
*Viola kauaensis* var. *kauaensis*	1	Kaua`i	*P.K. Higashino PKH 9633*	BISH
*Viola kauaensis* var. *kauaensis*	1	Kaua`i	*T.G. Lammers 5382*	BISH
*Viola kauaensis* var. *kauaensis*	1	Kaua`i	*H.F.J. Huber 20*	BISH
*Viola kauaensis* var. *kauaensis*	1	Kaua`i	*W.L. Wagner 5049*	BISH
*Viola kauaensis* var. *kauaensis*	1	Kaua`i	*K. Kajimoto 1*	CAU
*Viola kauaensis* var. *kauaensis*	1	Kaua`i	*K. Kajimoto 2*	CAU
*Viola kauaensis* var. *kauaensis*	1	Kaua`i	*K. Kajimoto 3*	CAU
*Viola kauaensis* var. *kauaensis*	1	Kaua`i	*K. Kajimoto 4*	CAU
*Viola kauaensis* var. *kauaensis*	1	Kaua`i	*K. Kajimoto 5*	CAU
*Viola kauaensis* var. *kauaensis*	1	Kaua`i	*H. St. John 1347*	DUKE
*Viola kauaensis* var. *wahiawaensis*	1	Kaua`i	*H. St. John 10845*	BISH
*Viola kauaensis* var. *wahiawaensis*	1	Kaua`i	*H.U. Stauffer 5911*	BISH
*Viola kauaensis* var. *wahiawaensis*	1	Kaua`i	*D.R. Herbst 2415*	BISH
*Viola kauaensis* var. *wahiawaensis*	1	Kaua`i	*B.C. Stone 1650*	BISH
*Viola kauaensis* var. *wahiawaensis*	1	Kaua`i	*L.H. MacDaniels 606*	BISH
*Viola kauaensis* var. *wahiawaensis*	2	Kaua`i	*C.N. Forbes 166.K* (Holotype)	BISH
*Viola kauaensis* var. *wahiawaensis*	1	Kaua`i	*C.N. Forbes 166.K* (Isotype)	BISH
*Viola vanroyenii*	3	Kaua`i	*P. van Royen 11733* (Holotype)	BISH

### Floral morphology

Only specimens of *Viola kauaensis* var. *kauaensis* from Kaua`i and *Viola kauaensis* var. *wahiawaensis* possessed open and mature flowers suitable for measuring petals. All specimens of *Viola vanroyenii* and *Viola kauaensis* from O`ahu possessed either cleistogamous flowers or flowers without fully developed petals. St. John (1989) included a three dimensional sketch of a chasmogamous flower and sketches of dissected floral organs in his description of the type of *Viola hosakae*. Due to the degraded nature of the petals in a fragment envelope of the type of *Viola hosakae* at BISH we were unable to reassess size and shape of floral organs from O`ahu. St. John’s (1989) measurements of floral organs from *Viola hosakae* are referenced in discussions of petal size.

Although no *Viola kauaensis* individuals with chasmogamous flowers were observed during the 2013 surveys on O`ahu, several unopened flowers were observed. In the field, it was not obvious if the flowers represented unopened chasmogamous flowers or fully developed cleistogamous flowers. One flower was collected and preserved in 70% ethanol (multiple flowers were not collected to reduce detrimental impact on the small population). The flower was rehydrated in distilled water prior to dissection. Floral organs were removed and attached to an archival slide. The size and shape of floral organs were compared to Skottsberg’s (1940) illustrations of cleistogamous and chasmogamous floral organs from Kaua`i individuals of *Viola kauaensis*.

## Results

### Field measurements

Site A contained approximately 70 individuals scattered throughout four isolated patches. The violets grow from a layer of exposed moss on heavily sloped areas (Figure 1). All violets in the area had a small stature, less than 5 cm in height above the moss layer. No conspicuous chasmogamous flowers were observed. Lamina dimensions in the field ranged from 2–16 mm in length to 2–15 mm in width. The average leaf lengths and widths were 11 and 11.5 mm, respectively. Despite a thorough search, no individuals of *Viola kauaensis* were observed at site B.

### Analyses

Data from herbarium specimens show *Viola kauaensis* populations from O`ahu possessed consistently smaller mean values of morphological and reproductive traits when compared to populations on Kaua`i (Table 2). Minimum ranges of morphological values from Kaua`i overlap with maximum values from the O`ahu individuals.

**Table 2.** Descriptive statistics for select traits quantified for varieties of *Viola kauaensis* and *Viola vanroyenii*. Mean and min-max values are provided. The table includes data from all herbarium specimens analyzed, not just those used for PCA. All measurements in mm.

Trait	*Viola kauaensis* var. *kauaensis* (Kaua`i)		*Viola kauaensis var. wahiawaensis*		*Viola kauaensis* (O`ahu)		*Viola vanroyenii*	
	mean	min-max	mean	min-max	mean	min-max	mean	min-max
Lamina length	30	11.5–58	36.33	21–52	17.35	8.5–24	6.94	5.5–9
Lamina width	36.37	12.5–77	34.08	14–45	17.27	8–26	8.36	6–11
Petiole length	58.94	9–220	103.17	15–180	33	4–80	8	5–17
Cauline stipule length	7.46	4–14	6.3	3–12	3.18	2–5	3.81	2.5–5
Cauline stipule width	2.75	1.5–5	2.2	1.5–3	1.55	1–2.5	1.62	1–2.5
Sepal length	6.65	5–11	6.86	6–8	4.05	2–6	3.8	3–4.5
Sepal width	2	1–4	1.91	1–3	1.31	0.5–2	1.75	1–3
Capsule valve length	11.1	4.5–17	15	15	7.4	5–9	6.43	5.5–8

**Figure 2.**
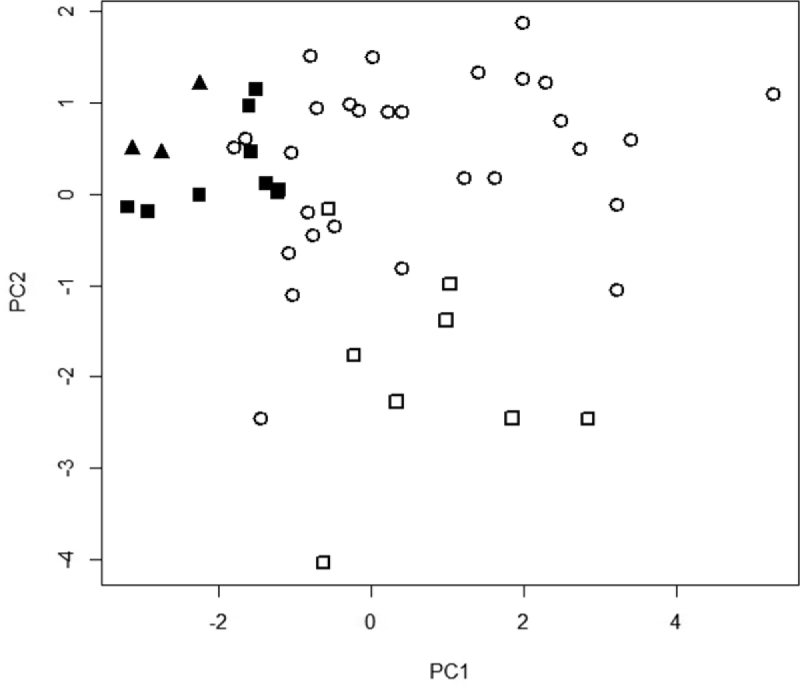
PCA Biplot of PC1 and PC2. Symbols: closed squares = *Viola kauaensis* var. *hosakae*; closed triangles = *Viola vanroyenii*; open circles = *Viola kauaensis* var. *kauaensis*; open squares = *Viola kauaensis* var. *wahiawaensis*.

The PCA yielded three principal components (PC) with a cumulative proportion of 0.8831 (Table 3). The first two PCs possessed eigenvalues greater than 1.0 and were retained for the construction of a biplot (Figure 2). The biplot depicts overlap between *Viola kauaensis* var. *wahiawaensis* and *Viola kauaensis*var. *kauaensis*. These two varieties are primarily separated along PC2, controlled by apex angle and base angle (Table 2). Individuals of *Viola kauaensis* from O`ahu cluster together but overlap slightly with individuals of *Viola kauaensis* from Kaua`i. *Viola vanroyenii* completely overlaps with individuals of *Viola kauaensis* from O`ahu. The O`ahu and Kaua`i populations of *Viola kauaensis* are primarily separated along PC1, controlled by lamina width (Table 3).

**Table 3.** Summary of PCA. Loadings for each variable are presented for the first three Principal Components.

**Variable**	**PC1**	**PC2**	**PC3**
Lamina length	0.47695396	-0.24381186	0.1296115
Lamina width	0.50188378	0.02178338	0.1166609
Petiole length	0.37770255	-0.23925298	0.5721113
Apex angle	0.08986523	0.61588137	0.4825464
Base angle	0.11415244	0.67522051	-0.1376558
Cauline stipule length	0.40548950	-0.06814306	-0.5148904
Cauline stipule width	0.43867994	0.20727565	-0.3540727
Proportion of variance	0.5362	0.2204	0.1266
Cumulative proportion	0.5362	0.7565	0.8831
Eigenvalue	3.75322839	1.54260388	0.88597604

### Floral morphology

Removal of sepals from the preserved flower (*Viola kauaensis* from O`ahu) indicated the presence of several withered petals and only two stamens with anthers. The stamens contained an elongated filament with anthers at their tip. The anther from one of the stamens was in direct contact with the stigmatic surface of the pistil. The style was also relatively short and curved towards the anther. All of these observations were consistent with Skottsberg’s (1940) illustrations of cleistogamous floral organs in *Viola kauaensis* specimens from Kaua`i.

## Discussion

Herbarium and field data suggest that the herbaceous *Viola* on O`ahu occupy a much more limited range of morphological variation than *Viola kauaensis* var. *kauaensis*. Individuals in the O`ahu population, like those individuals on Kaua`i, also produce cleistogamous flowers. The individuals on O`ahu demonstrate a fixed range of variation in the size of leaves, petioles, cauline stipules, and flowers. Although not evidenced in the PCA by contribution of base angle along PC1, individuals on O`ahu do not possess the extreme reniform leaf bases often observed in *Viola kauaensis* var. *kauaensis*. This may just be a trait that is exaggerated in larger leaves. Due to these variations, the O`ahu individuals are best treated at a distinct infraspecific rank within *Viola kauaensis*: *Viola kauaensis* var. *hosakae*.

**Figure 3.**
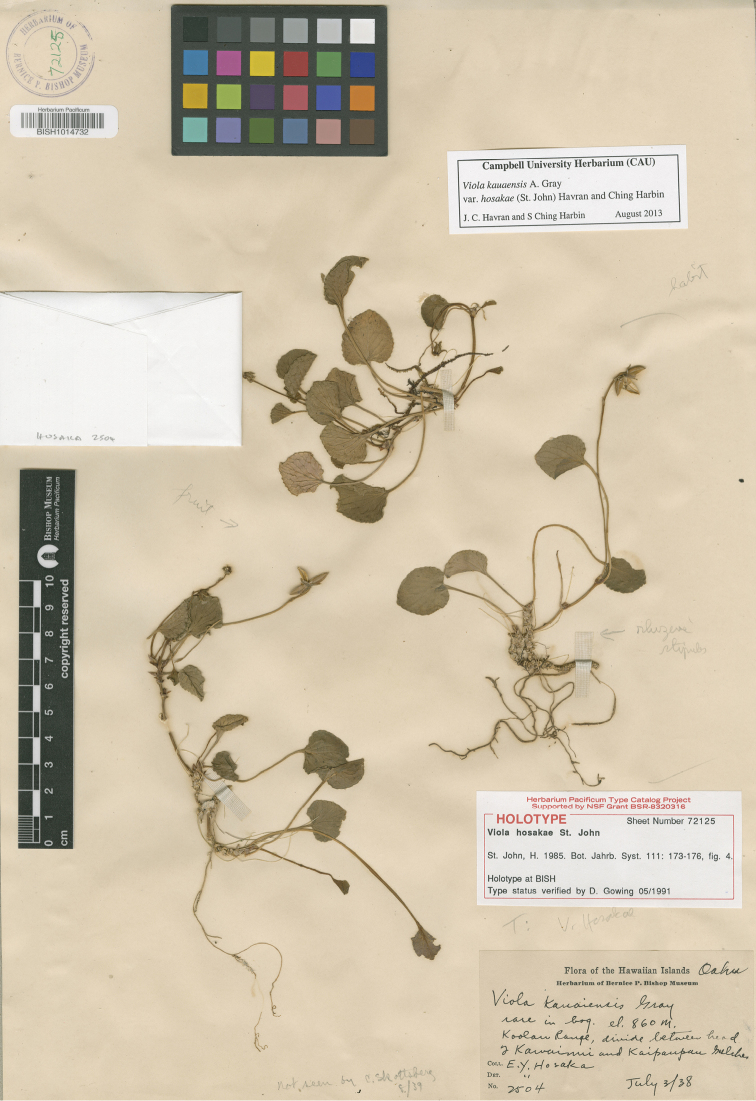
Type of *Viola kauaensis* var. *hosakae*.

Recently collected specimens on O`ahu represent a much smaller range of size than those individuals collected early in the 20^th^ century. Leaves of recently collected materials are considerably smaller than those in the type (Figure 3) and represent those individuals more distinct from *Viola kauaensis*var. *kauaensis* in the PCA biplot. *Viola kauaensis* has been collected from multiple sites on O`ahu, but is now known from just one population. The variety may have existed across the Ko`olau Mountains in a wide range of sizes, but now persists as a solitary population in the smaller extreme of leaf size. The reduction in range size may be associated with interaction with non-native species. The invasive grass *Axonopus fissifolius* (Raddi) Kuhlm has had a negative impact to summit plants on O`ahu and is found growing alongside *Viola kauaensis* var. *hosakae*. The action of ungulates along the summit area would also detrimentally impact the survivorship of the variety.

The varieties of *Viola kauaensis* on Kaua`i and O`ahu occupy different habitats. On Kaua`i *Viola kauaensis* var. *kauaensis* is distributed in the open bog and cloud forest margins of the high-elevation Alakai Swamp. In the bog environments, the species is usually distributed in hummocks of *Metrosideros polymorpha* Gaud., mosses, and lichen, while in bog margins the species can be found growing terrestrially or epiphytically in pockets of moss on tree stems. On O`ahu, the one population of *Viola kauaensis* var. *hosakae* contains at least four smaller subpopulations of 4-30 individuals scattered over an area of about 50 m^2^. Each subpopulation is distributed on a moderate to steeply sloping surface with individuals growing directly out of unsaturated exposed soil or from a thin layer of moss (Figure 1). This microhabitat description differs greatly from the typical habitat of *Viola kauaensis* var. *kauaensis* on Kaua`i (Wagner et al. 1999), especially with regard to the slope.

Havran et al. (2009) and Ballard (2000) included the O`ahu herbaceous violets in their phylogenies of the endemic Hawaiian *Viola*. In both studies of the Internal Transcribed Spacer (ITS) sequences, the O`ahu populations grouped closely with *Viola kauaensis* var. *kauaensis*. Neither study incorporated material from *Viola kauaensis* var. *wahiawaensis*. The O`ahu individual possessed four differences in the ITS sequence regions compared with the Kaua`i material. The variation is one of the largest seen when comparing interisland populations of conspecifics in the wet clade of Hawaiian violets. *Viola kauaensis* var. *hosakae* likely diverged from *Viola kauaensis* var. *kauaensis* following an interisland dispersal event from Kaua`i to O`ahu.

While it is likely that *Viola kauaensis* var. *hosakae* may have been derived through allopatric speciation, the relationship between *Viola kauaensis* and *Viola vanroyenii* is less clear. *Viola vanroyenii* falls within the range of morphological variation as *Viola kauaensis* var. *hosakae*, but outside the range of variation of *Viola kauaensis* var. *kauaensis* along PC1. Field observations by Steve Perlman (personal communication) indicate that *Viola vanroyenii* is sympatric with *Viola kauaensis* *kauaensis* on Kaua`i. *Viola vanroyenii* may represent *Viola kauaensis* var. *kauaensis* at the smaller extreme of its morphological variation, possibly as a result of harsh conditions at the summit area of Mt Waiale`ale. If more individuals are ever found, this relationship should be reevaluated.

## Taxonomic treatment

### Viola kauaensis A. Gray var. hosakae (H.St.John)

Taxon classificationPlantaeMalpighialesViolaceae

Havran & Ching Harbin comb. et. stat. nov.

urn:lsid:ipni.org:names:77140439-1

Figure 1B , 3 Additional figures: St. John (1989) Bot. Jarb. Syst. 111(2) 165–204 (Figure 4).

#### Basionym.

*Viola hosakae* H.St.John, Botanische Jarbücher für Systematik, 111(2), 173, 1989.

#### Type.

Hawai`i, O`ahu Island, Ko`olau Range, divide between head of Kawainui and Kaipaupau Gulches, rare in bog, 860 m elev., *E.Y. Hosaka 2504*; July 3, 1938 (holotype: BISH! Sheet no: 72125).

#### Description.

*Rhizomatous herb, rhizome* creeping rhizome stipules 1.5–3.0 mm long, 1–2 mm wide, often overlapping and scaly in appearance; vertical stems produced from rhizome, internodes on vertical stem longer than on rhizome, stipules 2.0–5.0 mm long, 1.0–2.5 mm wide. *Flowers* solitary on terminal peduncle, flower subtended by opposite pair of small linear bracts on peduncles. *Chasmogamous flower* characteristics as in St. John (1989): dorsal sepal 5 × 1.4 mm, elliptic; lateral sepal 4.5 × 1.4 mm, obovate elliptic; ventral sepal 5.6 × 1.4 mm, lance elliptic; dorsal petals 15 × 3.3 mm, with a 4 mm claw and an elliptic blade; lateral petals 14 × 2.6 mm, with a broad 4 mm claw and an elliptic blade; ventral petal 16 mm long, with a curved 6 mm channeled claw, and an elliptic blade that is 5 mm wide; dorsal stamen 3.9 mm long, filament 0.5 mm long, stout, oblique, anther 2.5 mm long, narrowly obovoid ellipsoid, sterile tip 1.3 mm long, ovate; lateral stamen 3.9 mm long, filament 0.5 mm long and broad, anther 2.3 mm long, narrowly cuneoid, sterile tip 1.3 mm long, ovate, acute; ventral stamen 3.6 mm long, filament 0.5 mm long and wide, anther 2.3 mm long oblanceoloid, sterile tip 1.5 mm long, lanceolate, nectary 1.5 mm high, 0.8 mm wide, arcuate oblong, basal; pistil 2.8 mm long; style 1 mm long; stigma discoid, divergent at 45°; chasmogamous flowers not seen (see methods). *Cleistogamous flowers* with linear sepals 5, green, 5–6 mm long, 1 mm wide, bases auriculate, apices acuminate, enclosing all other floral organs; petals 5 or fewer, up to 3 mm long, 1 mm wide, white, with withered appearance; stamens 2, 1.5 mm long, filament 1 mm long, anthers 0.5 mm long and at end of filament, anther in direct contact with stigmatic surface of pistil; pistil 2 mm long, ovary 1.5 mm long, style 0.5 mm long, curved at approximately 180˚ towards ovary. *Fruit* a capsule, capsule valves 7–9 mm long.

#### Distribution.

Hawaiian Islands, O`ahu: Poamoho summit region of Ko`olau Mountains.

#### Specimens Examined.

**Hawaiian Islands:** O`ahu: Laie, 19 Dec 1937, *Hosaka 1927* (BISH); Main divide, crest of Ko`olau Mts, above Kaipapau Gulch, 31 May 1937, *Fosberg 13973* (BISH); Main divide, crest of Ko`olau Mts, above Kaipapau Gulch, 24 Jul 1937, *Fosberg 14229* (BISH); About one half mile south of Poamoho trail along the Ko`olau Summit trail, 20 May 2013,*Havran 2013.4* (BISH); About one half mile south of Poamoho trail along the Ko`olau Summit trail, 20 May 2013, *Havran 2013.5* (BISH); Ko`olau Mt summit, on small hill at Puu Pauau, between Poamoho and Schofield-Waikane trail, on west side of Summit trail, about 50 ft. from trail, 12 Mar 1995, *Perlman 14704* (PTBG); Ko`olau Mts. Between summit of Poamoho trail and Schofield trail, along summit crest on small hill, about 0.5 miles south of cabin, 7 Sep 1987, *Perlman 6456* (PTBG).

#### Conservation status.

*Viola kauaensis* var. *hosakae* appears very rare on O`ahu. Despite frequent and thorough conservation work by multiple organizations in the summit area of the Ko`olau Mountains, only one population of the variety is known to exist. The population is threatened by grazing ungulates. In addition, island tropical montane environments, like the ones harboring *Viola kauaensis* var. *hosakae*, are incredibly susceptible to global climate change (Loope and Giambelluca 1998).

*Viola kauaensis* var. *hosakae* is best classified as Critically Endangered (CR) according to the IUCN Red List Criteria as it meets the following criteria: B. Area of occupancy less than 10 km^2^, number of populations = 1, and continuing decline inferred from extent of occurrence and area of occupancy as indicated from herbarium records and personal communication; C. Number of mature individuals less than 250 and an estimated continuing decline (C2) with less than 50 mature individuals in each subpopulation (C2i).

The Plant Extinction Prevention Program (PEPP) branch on O`ahu will work to preserve this taxon on by collecting and germinating seeds when possible. Efforts are underway to enclose the population within an ungulate fence by the end of 2014. An additional population can be started with propagules from the extant population. The cleistogamous reproduction of the variety should help to facilitate seed production in the absence of pollinators at a new location.

### Key to the varieties of *Viola kauaensis*

**Table:** 

1	Leaf base cuneate	*Viola kauaensis* var. *wahiawaensis*
–	Leaf base truncate to cordate	2
2	Leaf base truncate to deeply cordate; lamina 13 – 77 mm wide, generally widest in 7 third of lamina, sepals 5-11 mm long; stipules subulate to lanceolate, margins sparsely serrate	*Viola kauaensis* var. *kauaensis*
–	Leaf base rounded, truncate, or shallowly cordate; lamina 8-26 mm wide, generally widest in middle of lamina, sepals 2–6 mm long; stipules linear to lanceolate, margins dentate to erose	*Viola kauaensis* var. *hosakae*

## Supplementary Material

XML Treatment for Viola kauaensis A. Gray var. hosakae (H.St.John)

## Additional specimens examined

*Viola kauaensis* var. *kauaensis*: **Kaua`i**: Alakai Swamp Trail, 27 Dec 1956, *Stone 1552* (BISH); Wai`ale`ale, 28 Jun 1925, *Brown 1247* (BISH); Kaholuamanu, no date, *Lydgate s.n*. (BISH); Waimea Drainage Basin W side, 3 Jul 1917, *Forbes 1135K* (BISH); Waimea below Kokee on Kilohaua, 27 Oct 1922, *Skottsberg 939* (BISH); Kokee, “Waineke Swamp”, 28 Jun 1926, *Degener 3351* (BISH); Lehua Makanoe, bogs of Waimea, 3 Mar 1909, *Rock 2124* (BISH); Kaholuamanu, no date, *Rock 2121* (BISH); Lehua Makanoe, 8 Mar 1909, *Rock 2130* (BISH); Alakai Swamp Drainage Basin, W Side, 3 Jul 1917, *Forbes 906K* (BISH); Alakai Swamp, 25 Apr 1372, *Herbst 2388* (BISH); Alakai Swamp in bog areas and on route to Wai`ale`ale, 1 Sep 1977, *van Royen 11708* (BISH); Waimea District, Waimea AHU, Alakai Swamp Tr, 16 May 1981, *Higashino PKH 9679* (BISH); Waimea District, Waimea AHU, Alakai Swamp Tr, 12 May 1981, *Higashino PKH 9643* (BISH); Alakai Swamp, Aipoinui bog, 21 Sep 1985, *Takeuchi s.n*. (BISH); Alakai Swamp, north of Kilohana, near Wainiha rim, TNC survey of Hono O Napali NAR, transect 10, 18 May 1988,*Perlman 9224* (BISH); Alakai Swamp, Aipoinui bog, 19 Jan 1952, Degener 21747 (BISH); Napali, Kona Forest Reserve NW end of Alakai Swamp, 27 Dec 1930, *St. John 10753* (BISH); Kaholuamanu Lehua Makanoe, E of Waialae, Sep 1909, *Forbes 406K* (BISH); Below Mt. Wai`ale`ale, Mar 1932, *Rogers 1* (BISH); Kaholuamanu, 3 Mar 1909, *Rock 2131* (BISH); Mts above Waimea, no date, *Mann s.n*. (BISH); Lehua Makanoe, 16 Sep 1909, *Rock 5715* (BISH); Kokee, Halemanu, Dec 1951, *Degener 21477* (BISH); Alakai Trail NE of crossing of second n fork of Kawaikoi Stream at B.M. 3638, 25 Dec 1947, *St. John 23038* (BISH, DUKE); Kilohana from the rain gage bog, 31 Jul 1983, *Takeuchi Alakai_130a* (BISH); Waimea District, Waimea AHU, Alakai Swamp Tr, 12 May 1981, *Higashino PKH 9633* (BISH); Waimea District, Waimea AHU, Alakai Swamp Along trail betw Pihea and Kilohana, 30 Aug 1983, *Lammers 5382* (BISH); Alakai Swamp, 21 Aug 1964, *Huber 20* (BISH); Kokee State Park along Alakai Swamp Trail from jct with Pihea Trail to Kilohana, 30 Aug 1983, *Wagner 5049* (BISH); Mt. Wai`ale`ale, 200 m from summit, 14 Nov 2012, *Kagimoto 1* (CAU); Mt. Wai`ale`ale, 200 m from summit, 14 Nov 2012, *Kagimoto 2* (CAU); Mt. Wai`ale`ale, 200 m from summit, 14 Nov 2012, *Kagimoto 3* (CAU); Mt. Wai`ale`ale, 200 m from summit, 14 Nov 2012, *Kagimoto 4* (CAU); Mt. Wai`ale`ale, 200 m from summit, 14 Nov 2012, *Kagimoto 5* (CAU); Kaua`i, 1838, *Wilkes s.n*. [HOLOTYPE] (US).

*Viola kauaensis* var. *wahiawaensis*: **Kaua`i**: Waimea Bog, 28 Dec 1956, *Wolford 397* (BISH); Wahiawa, Kahili Swamp, 29 Dec 1930, *St. John 10845* (BISH); Wahiawa, Kahili Bog, 24 Dec 1933, *St. John 13555* (BISH); Kola District Wahiawa Swamp near headwaters of Wahiawa Stream, 24 Apr 1964, *Stauffer 5911* (BISH); Wahiawa Swamp, Aug 1909,*Forbes 166.K* [Holotype] (BISH); Wahiawa Bog, 22 May 1972, *Herbst 2415* (BISH); Wahiawa Bog (Kanaele Swamp) W of Kahili Mt, 2 Jan 1957, *Stone 1650* (BISH); Bog above Wahiawa, 8 Feb 1927, *MacDaniels 606* (BISH); Wahiawa Mts, no date, *Lydgate s.n*. (BISH); In Wahiawa Bog (Kanaele Swamp), 22 Dec 1983, *Wagner 5202* (BISH); Wahiawa Swamp, Aug 1909, *Forbes 166.K* [Isotype] (BISH); Wahiawa Swamp, Aug 1909,*Forbes 166.K* [Isotype] (BISH); Koloa District, Wahiawa Swamp near headwaters of Wahiawa Stream, 24 Apr 1964, *Stauffer 5911* (DUKE).

*Viola vanroyenii*: **Kaua`i**: Wai`aleale summit area, Alakai Swamp, 3 Sep 1977, *van Royen 11733* [Holotype] (BISH).
